# Inquiries Concerning Tubercular Diseases

**Published:** 1834

**Authors:** 


### V. Inquiries Concerning Tubercular Diseases.

We have received from Dr. James Jackson, jr. of Boston, a printed circular of Dr. Clark, of London, containing the following inquiries relative to tubercular diseases, and especially tubercular consumption. Dr. C. is a physician of learning and eminence, already favorably known as a writer on the Influence of Climate, and the subject of his present inquiry is one of deep interest. We take great pleasure in presenting his queries to our reader, and beg leave to add the expression of a hope, that several of them will be found qualified and willing to aid in the inquiry. Their communications may either be sent (post paid) to the Editor of this Journal, or by private hand to the post office in New-York, directed—"James Clark, M.D. Author of a Treatise on the Influence of Climate, London, Great Britain." Dr. Clark is desirous of receiving early replies.

1. Do you consider Tuberculous Consumption, and Tuberculous or Scrofulous diseases generally, more or less prevalent among the *opulent classes** of society at the present time than† years ago?

2. Every medical man of observation must be more or less acquainted with the state of health of three generations:—What is the comparative state of health of these respectively?—Is the general standard of health higher or lower—and are Tuberculous‡ diseases more or less prevalent in the rising generation, than in the two preceding generations?

*By the opulent class are meant all those enjoying the comforts of life.

†The space to be filled up according to the extent of your experience.

‡The terms Tuberculous and Scrofulous are used synonymously.

3. To what circumstances do you attribute any change which you may have remarked in the state of health of the more opulent classes?

4. What do you consider the relative prevalence of Scrofulous diseases among the *lower orders** of society at the present moment, and† years ago.

5. Do your observations refer more especially to any particular class of people?

6. To what circumstances in the condition or habits of the people do you attribute any change in the state of their health, which you may have observed?

7. Have any particular occupations appeared to you to give rise to Tuberculous Consumption, or to Scrofula, in a marked degree?—If so, specify them.

8. Have you remarked any occupations which give an evident, comparative, exemption from Consumption or Scrofula?—If so, specify them.

9. Have you observed Tuberculous diseases to prevail more or less in particular localities?—If so, specify them.

10. Have you observed Tuberculous diseases to prevail very generally among spirit-drinkers?

11. What do you consider the chief causes of Scrofulus diseases generally, and of Consumption more particularly, among the working classes?

12. What is the prevailing form which Scrofulous disease assumes, and what organs or structures does it chiefly affect, in the district under your observation?

13. In the course of your experience, have you observed any change in the prevailing type or forms of Tuberculous diseases? Are the internal or external organs, comparatively, more or less frequently affected now than they were formerly?

14. Have you observed any difference of character between the Tuberculous diseases of the opulent and the working classes generally? or between the diseases of *the children* of these two classes more particularly?

15. Have you remarked a connection between any particular derangement of health of the parents, and Scrofula in the offspring.—If so, specify it.

16. Have you observed any derangement in the general health, or any disorder of particular organs of individuals to precede the appearance of Tuberculous diseases in them?—If so, specify such general or local affection.

17. At what period of life have you observed Tuberculous Consumption most frequently to make its appearance?

18. Have you observed any particular diseases to affect persons of a Scrofulous habit, after they have reached their 50th year?

*Under this title, include all those who gain their livelihood by bodily labor chiefly.

†The space to be filled up according to the extent of your experience.

